# Efficacy of different DNA and MVA prime-boost vaccination regimens against a Rift Valley fever virus (RVFV) challenge in sheep 12 weeks following vaccination

**DOI:** 10.1186/s13567-018-0516-z

**Published:** 2018-02-21

**Authors:** Gema Lorenzo, Elena López-Gil, Javier Ortego, Alejandro Brun

**Affiliations:** Instituto Nacional de Investigación y Tecnología Agraria y Alimentaria (INIA), Centro de Investigación en Sanidad Animal (CISA), Valdeolmos, 28130 Madrid, Spain

## Abstract

The aim of this work was to evaluate the immunogenicity and efficacy of DNA and MVA vaccines encoding the RVFV glycoproteins Gn and Gc in an ovine model of RVFV infection. Adult sheep of both sexes were challenged 12 weeks after the last immunization and clinical, virological, biochemical and immunological consequences, were analyzed. Strategies based on immunization with homologous DNA or heterologous DNA/MVA prime-boost were able to induce a rapid in vitro neutralizing antibody response as well as IFNγ production after in vitro virus specific re-stimulation. In these animals we observed reduced viremia levels and less clinical signs when compared with mock-immunized controls. In contrast, sheep inoculated with a homologous MVA prime-boost showed increased viremia correlating with the absence of detectable neutralizing antibody responses, despite of inducing cellular responses after the last immunization. However, faster induction of neutralizing antibodies and IFNγ production after challenge were found in this group when compared to the mock vaccinated group, indicative of a primed immune response. In conclusion, these results suggest that vaccination strategies based on DNA priming were able to mount and maintain specific anti-RVFV glycoprotein immune responses upon homologous or heterologous booster doses, warranting further optimization in large animal models of infection.

**Electronic supplementary material:**

The online version of this article (10.1186/s13567-018-0516-z) contains supplementary material, which is available to authorized users.

## Introduction

Rift Valley fever (RVF) is an emerging zoonosis of ruminants caused by a phlebovirus transmitted by several mosquito species present in both tropical and temperate settings [1]. The virus can infect and replicate in wild and domesticated ruminants resulting in high rates of mortality and abortion in newborn lambs and gestating ewes respectively [2]. As a member of the novel *Bunyavirales* order, family *Phenuiviridae*, Rift Valley fever virus (RVFV) is composed of a tripartite ssRNA(−) genome, comprising large (L), medium (M) and small (S) segments. The L-segment encodes a RNA-dependent RNA polymerase (RdRp) responsible of transcribing and replicating the incoming viral genome. The M-segment encodes two structural glycoproteins (Gn and Gc) responsible of cell-attachment and fusion being the main targets for neutralizing antibodies, as well as two accessory proteins: a 13–14 kDa non-structural anti-apoptotic protein (termed NSm′ and NSm, respectively) and a 78 kDa protein, suggested to be incorporated into viral particles when expressed in mosquito cells [3, 4]. The synthesis of M segment-encoded proteins relies on a ribosomal leaky scanning mechanism and differential use of 5 putative in-frame AUG codons to initiate translation [3]. The S segment encodes two genes in an ambisense orientation: the viral nucleoprotein N, that associates with the viral ssRNA(−) to form the nucleocapsid, and the multifunctional, virulence-associated, non-structural protein NSs [5, 6]. In the African continent, RVFV causes recurrent disease outbreaks in both humans and livestock following abnormally high wet seasons. The disease is also prevalent outside continental Africa since year 2000 when it was spread to the Arabian Peninsula [7] and Indian Ocean islands [8–10]. Trade and globalization in the context of a global climate warming might be key drivers for virus introduction in the future, increasing the probabilities of virus dissemination and maintenance in European countries considering the presence of indigenous competent mosquito species [10, 11]. These concerns aimed the development of improved diagnostic methods as well as safer RVF vaccines for use in ruminants since current licensed RVF vaccines do not meet European safety standards. Two vaccines have been traditionally used to control disease outbreaks in South Africa: a formalin inactivated vaccine [12] and a live attenuated virus strain [13]. Both vaccines have disadvantages such as low immunogenicity and potentially adverse side effects, respectively. A new live-attenuated vaccine termed “Clone 13”, now licensed for use in several African countries, is very immunogenic and highly effective in protection but may not be fully recommended for vaccination of pregnant animals since it has been reported recently to cause malformations and stillbirths when used at high doses [14]. In addition, clone 13 appears to be able to replicate in competent mosquito species [15, 16]. Up to date, virtually, most of the available vaccine technologies have been tested for efficacy against RVFV infection both in laboratory models or in large ruminants short after immunization [17]. Besides veterinary vaccines, safe RVF vaccines for humans may be demanded in the future for personnel at risk, including farmers, veterinaries and/or medical personnel. DNA vaccines provide a safer alternative to the use of live attenuated vaccines but they face the hurdle of inducing weak immune responses in large animal hosts, in spite of their proved efficacy in laboratory rodents [18]. In a previous work we reported that a single immunization of sheep with a purified recombinant modified vaccinia virus Ankara encoding the RVFV glycoproteins Gn and Gc (rMVA-GnGc) was able to reduce the viral excretion after challenge but did not prevented clinical display nor viremia [19]. In this work we have extended these observations to the use of prime-boost approaches using both DNA and/or recombinant MVA expressing Gn and Gc RVFV glycoproteins. Our results show that both the homologous DNA and the heterologous prime-boost (DNA + MVA) strategies were able to reduce clinical signs and viremia in adult sheep challenged long after immunization. These results indicate the ability of these vaccine strategies to elicit memory responses in sheep.

## Materials and methods

### Vaccines and challenge virus

The MVA vaccine was generated by homologous recombination within infected chicken embryo fibroblast (CEF) cells with a recombinant plasmid encoding GFP as marker and the RVFV-MP12 glycoprotein GnGc sequence (accession # DQ380208.1) in a bi-cistronic expression cassette as described [20]. The virus was grown in permissive DF-1 cells (ATCC# CRL-12203, American Type Culture Collection, Manassas, VA, USA), the supernatants and lysed cells harvested and virus pelleted by ultracentrifugation through 36% sterile sucrose cushion. Titration of concentrated vaccine virus was performed in DF-1 cell monolayers. Both vaccine virus and challenge virus stocks were stored at −80 °C until use. The plasmid DNA vaccine used here (pCMV-GnGc) was constructed as described previously [21] and contains the MP12 strain GnGc ORF specified by the fourth in frame initiation codon (nts 411–413), under control of the human immediate early CMV promoter.

The South African virulent RVFV 56/74 isolate used for sheep challenge studies was kindly provided by the Agricultural Research Council-Onderstepoort Veterinary Institute (South Africa). This virus strain had been isolated from cattle [22] and propagated in chicken embryo-related cells and MDBK cells. Two additional passages in BHK-21 (ATCC# CCL-10) cells were performed to generate a larger virus stock [23]. For the purposes of the present experiment the virus was further propagated in C6/36 cells. This virus stock was named 56/74_INS [24]. TCID_50_ titers of this stock were determined on Vero cells by the method of Reed and Muench [25].

### Experimental design, clinical records and sampling procedure

Animal experiments were approved and authorized by the Biosafety Committee and the Ethical and Animal Welfare Committee of INIA, in accordance with the EU directive 2010/63/EU. A total of twenty lambs from a Colmenareña sheep flock of both sexes, aged 12 weeks at the time of vaccination, were used. Animals were immunized in experimental farm at Department of Animal Reproduction (INIA) and then moved to the BSL-3 facility at CISA 1 week prior to challenge with RVFV. Lambs were fed following procedures used in conventional farms, with water supply provided ad libitum.

The lambs were distributed into four experimental groups of *n* = 5. The first group, was immunized with two doses of 10^8^ plaque-forming units (pfu) of the MVA-GnGc vaccine (dubbed “MVA vaccine” group). A second group received three serial doses of 400 µg of plasmid pCMV-GnGc mixed 1:1 with Lipofectin (Invitrogen, Carlsbad, CA. USA) reagent in a final volume of 0.5 mL (dubbed “DNA vaccine” group), and a third group was immunized with two serial doses of pCMV-GnGc plus a booster dose of MVA-GnGc (named “DNA + MVA vaccine” group). The last group (infection control) received only saline solution. The MVA vaccine was inoculated subcutaneously (sc) into the right subscapular skin fold using a 25 g needle injection, while the DNA doses were administered by the intramuscular (im) route (left hind limb). Repeated doses of vaccination were administered every 2 weeks. Before and after injection, the injection site was disinfected using 70% ethanol.

Twelve weeks after the last immunization the lambs were sc challenged with 10^5^ TCID_50_ of the RVFV strain 56/74_INS [24]. Clinical signs, including rectal temperature and behaviour, were monitored and recorded daily until the end of the experiment. The extent of disease was quantified using criteria and evaluation scores approved by the Ethical and Animal Welfare Committee of INIA. Clinical signs for scoring the extent of morbidity were anorexia, nasal and/or ocular discharge, diarrhea, prostration and weakness. The fever threshold was set to ≥ 40.3 °C based on the mean plus three standard deviations of the rectal temperatures recorded in the 20 sheep just before challenge.

Blood and serum samples were obtained from each animal on the day of challenge, daily during the first week after challenge, and at days 8, 12, 15 and 18 post-challenge, for virological, biochemical and immunological analysis. After collection, blood samples in EDTA containing vessels were directly frozen to −80 °C for further processing.

### Virus titration in blood samples

Blood samples recovered in anticoagulant vessels were lysed in sterile distilled water and a proportional volume of tenfold concentrated sterile PBS was added to restore physiological pH and isotonic conditions, resulting in a final blood dilution of 1:20. 100 µL of twofold dilutions (starting at 1:40) were added to Vero cells (ATCC# CCL-81) cultured in 96-well plates in quintuplicate. After incubation at 37 °C in a 5% CO_2_ atmosphere for 90 min, the supernatants were withdrawn under vacuum aspiration, the cells washed twice and replaced with fresh media (DMEM supplemented with 2% FBS). After incubation for 6 days the cells were fixed and stained with 10% formaldehyde and 2% crystal violet solution, respectively. TCID_50_ end point titers were calculated by the Reed and Muench method [25].

### Biochemical determinations in blood samples

Alanine aminotransferase (ALT), aspartate aminotransferase (AST), serum albumin (ALB), alkaline phosphatase (ALP), gamma-glutamyltransferase (GGT), lactate dehydrogenase (LDH), total bilirubin (BIL), total protein (TP) and blood urea nitrogen (BUN) levels in sheep sera collected at different time points post-infection were measured in a Saturno 100 analyzer (Crony Instruments, Rome, Italy) using specific reagents according to the manufacturer’s instructions (SpinReact, Vall D’En Bas, Spain). Normal ranges for ovine species were used as reference values [26].

### ELISA and virus neutralization assays

Anti-RVFV glycoprotein Gn and Gc antibodies were detected using an indirect ELISA using purified recombinant antigens (1 μg/well). Purified Gn and Gc proteins were a generous gift of Dr. Jeroen Kortekaas (WVBR, Lelystad). Sheep sera were diluted to 1/100 prior to incubation. Detection of immune complexes was performed using anti-sheep IgG (H+L)-HRPO conjugates using TMB as substrate. Absorbance values were determined at 450 nm by an automated reader (BMG, Labtech, Offenburg, Germany). Neutralizing antibody titers were determined by plaque neutralization reduction test (PRNT_80_). Briefly, heat inactivated serum samples were serially diluted (twofold), starting at a dilution of 1:2.5 in DMEM medium containing 2% FBS, mixed with an equal volume of medium containing 10^2^ pfu of a stock of the MP-12 RVFV strain. After 1 h incubation at 37 °C, this mixture was added to Vero cell monolayers seeded in 12-well culture plates. After 1 h incubation, the cells were washed twice with medium and layered with 1% carboxy methyl cellulose (CMC; Sigma-Aldrich, St Louis, MO, USA) in DMEM supplemented with 10% bovine fetal calf serum. After 3 days incubation at 37 °C, cells were fixed with 10% formaldehyde and stained with a 2% crystal violet solution. The neutralization titer of each sample was defined as the reciprocal of the highest serum dilution resulting in a reduction of 80% of plaques.

### Isolation of cells from peripheral blood

Upon extraction, blood was first diluted into Acid Citrate Dextrose solution (ACD) using 4:1 (vol/vol) blood/ACD and centrifuged 5 min at 800 × *g*. Next, plasma fractions were removed and erythrocytes lysed by adding 0.83% NH_4_Cl solution. White blood cells were pelleted and subjected to several cycles of centrifugation and dilution in phosphate buffered saline (PBS) to remove stromal bodies and platelets. After a last wash the remaining cells were adjusted to a concentration of 5 × 10^7^ cells/mL in complete culture medium (RPMI 1640, 2 mM l-Glutamine and 10% fetal bovine serum).

### Interferon gamma enzyme linked immuno spot assay (ELISPOT)

Peripheral blood mononuclear cells (PBMC) were maintained in complete cell culture medium. 96-well MAIPS45 Immobilon-P filter plates (Merck Millipore, Billerica, MA, USA) were coated overnight at 4 °C with 2 µg/mL of mouse anti-bovine IFN-gamma antibody (clone MT17.1.; Mabtech, Stockholm, Sweden). Wells were washed and blocked with complete medium for 1 h at 37 °C. Blocking buffer was removed and a concentration of 5 × 10^5^ cells in 100 µL per well were incubated at 37 °C for 18 h with either 5 mg/mL Concanavalin A (Sigma Aldrich), purified RVFV MP12 strain grown in Vero cells or a non-infected Vero-cell extract (negative control). The plates were then washed extensively with distilled water and PBS and incubated with 0.25 µg/mL of biotinylated anti- IFNγ antibody (Mabtech clone MT307) for 2 h at room temperature. Afterwards, plates were washed with PBS and 100 µL of horseradish peroxidase (HRPO)-labeled streptavidin (1/1000 dilution in PBS; Becton-Dickinson, Franklin Lakes, NJ, USA) added to each well and incubated at room temperature for 1 h. Spots were visualized by the addition of 3-amino-9-ethylcarbazole Elispot substrate (Becton–Dickinson) and counted under magnification lens. Control wells with medium alone or with phytohemagglutinin (Sigma-Aldrich) at a final concentration of 5 µg/mL were also included.

### Detection of IFNγ in plasma samples by capture ELISA

96 well Costar plates (Sigma-Aldrich)) were coated with 2 µg/mL of mAb anti-bovine interferon-gamma (clone MT17.1, Mabtech). 100 µL of heparinized blood, collected at different time points before and after challenge from each animal were incubated in multiwell plates with purified RVFV MP12 strain extract or non-infected Vero cell extract (negative control) during 48 h. Next, the plates were centrifuged and plasma was collected and kept at −80 °C until use. 50 µL of plasma from each time point sample was assayed in the capture ELISA. After washing steps biotinylated anti bovine IFNγ (clone MT307, Mabtech) was incubated. Streptavidin-HRPO (Becton-Dickinson) was used for detection of immunocomplexes upon addition of TMB peroxidase substrate (Sigma-Aldrich). Absorbance values were determined at 450 nm by an automated reader (BMG, Labtech).

### Statistical analyses

Statistical comparisons were calculated using the GraphPad 6.0 software (La Jolla CA, USA). The Wilcoxon–Mann–Whitney two-sample rank-sum test was applied for comparisons between two groups.

## Results

### Clinical findings in sheep

**Figure 1:**
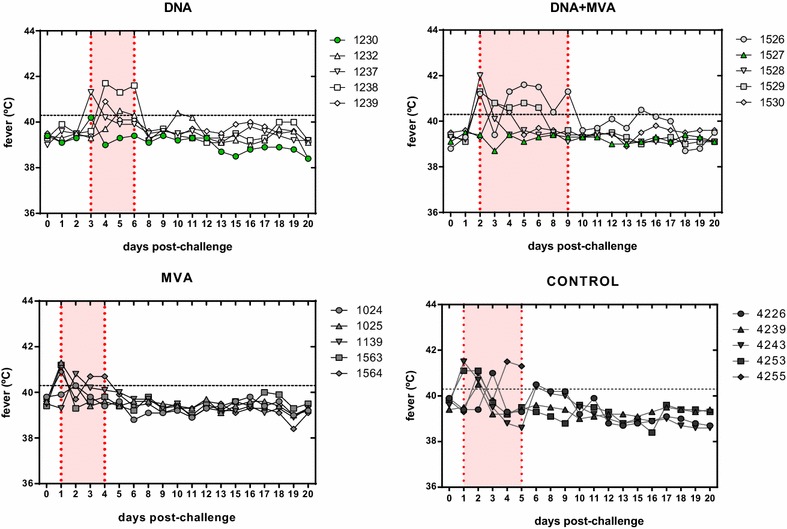
**Pyrexia in sheep after RVFV 56/74 challenge.** Rectal body temperatures in degrees Celsius (°C) were recorded daily during the experimental period. Fever was defined as a temperature above 40.3 °C (dashed line) based on the mean plus three standard deviations of individual temperatures recorded in all sheep just before challenge. The graphs display individual rectal temperatures sampled at similar times of sheep from the different vaccine groups. Shadowed areas show the pyrexic period of each group. Sheep with no fever are highlighted in green symbols.

After immunization with the different vaccines no clinical display nor adverse effects were noticed in any sheep. However, upon challenge with the RVFV 56/74 virus morbidity become apparent in all experimental groups. Likewise, fever was detected in all groups albeit differences in the time to onset, duration and number of animals affected were observed (Figure 1 and Table 1). All animals from the control group developed fever between 1 and 3 days post-challenge and two sheep displayed a later mild onset of fever by day 6 (#4226 and #4243). In this group one sheep (#4255) died at day 5 post-challenge after a sudden fever onset at day 4 post-challenge. At post-mortem examination sheep #4255 showed characteristic hepatic focal necrosis (not shown). All animals from the MVA group displayed a shorter peak of fever between 1 and 2 days after challenge, and by days 3–4 only one sheep (#1564) showed fever. In both DNA and DNA + MVA vaccinated groups at least one animal was found not febrile along the experiment, but fever was more persistent in two animals from the DNA + MVA group (#1526 and #1529). In contrast, a clear delay in the onset of fever was observed in the sheep from the DNA vaccine group. To quantify the extent of morbidity among the groups, a clinical score was applied based on the number of different clinical signs observed (Additional file 1). To do this, clinical signs showed for each animal each day were recorded daily after challenge. The clinical signs observed were: anorexia, nasal and/or ocular discharge, diarrhea, prostration and weakness. Despite all groups displayed sick animals some differences were found since animals vaccinated with DNA or the combination of DNA and MVA showed a delayed onset of clinical signs as well as a shorter clinical window (of 4 and 2 days, respectively) when compared to the control or MVA groups, both showing earlier clinical signs for 6 consecutive days.

**Table 1:** **Summary of clinical and virological findings in experimental vaccine groups**

Vaccine group	Sheep number	Fever^a^ (Y/N)	Fever onset^b^ (day)	No. days fever^c^	Max temp (day)^d^	Viremia (Y/N)^e^	Viremia onset (day)^f^	No. days viremia^g^	Max titer (logTCID_50_)^h^
Control	4226	Y	3	2	41.0 (3)	Y	2	3	2.9
	4239	Y	2	1	40.5 (2)	Y	2	1	2.5
	4243	Y	1	3	41.5 (1)	Y	2	2	4.2
	4253	Y	1	2	41.1 (1&2)	Y	2	2	3.1
	4255	Y	2	3	41.5 (4)	Y	2	4	5.6
DNA (3X)	1230	N	n/a	0	n/a	N	n/a	n/a	n/a
	1232	Y	5	3	40.5 (6)	N	n/a	n/a	n/a
	1237	Y	3	1	41.3 (3)	Y	3	2	3
	1238	Y	4	3	41.7 (4)	Y	4	1	2.3
	1239	Y	4	1	40.9 (4)	N	n/a	n/a	n/a
MVA (2X)	1024	Y	2	1	40.3 (2)	Y	2	2	3.6
	1025	Y	1	2	41.3 (1)	Y	2	2	4.2
	1563	Y	1	1	41.2 (1)	Y	2	2	4
	1564	Y	1	3	40.9 (1)	Y	2	4	4.8
	1139	Y	2	1	40.8 (2)	Y	2	2	4
DNA + MVA	1526	Y	2	7	41.6 (5)	Y	2	2	3.4
	1527	N	n/a	0	n/a	N	n/a	n/a	n/a
	1528	Y	2	1	42.0 (2)	N	n/a	n/a	n/a
	1529	Y	2	5	41.3 (2)	Y	2	2	3.6
	1530	Y	3	2	40.8 (3)	N	n/a	n/a	n/a

^a^Detection of rectal temperature ≥ 40.3 °C.

^b^Day after challenge in which fever is detected for first time.

^c^Number of days in which temperature ≥ 40.3 °C is detected.

^d^Maximum temperature detected and day.

^e^Detection of virus in blood samples.

^f^Day post-challenge at which viremia is detected for first time.

^g^Number of days in which viremia is detected.

^h^Maximum viremia titer post-challenge.

### Virus detection and titration in sheep blood samples

**Figure 2:**
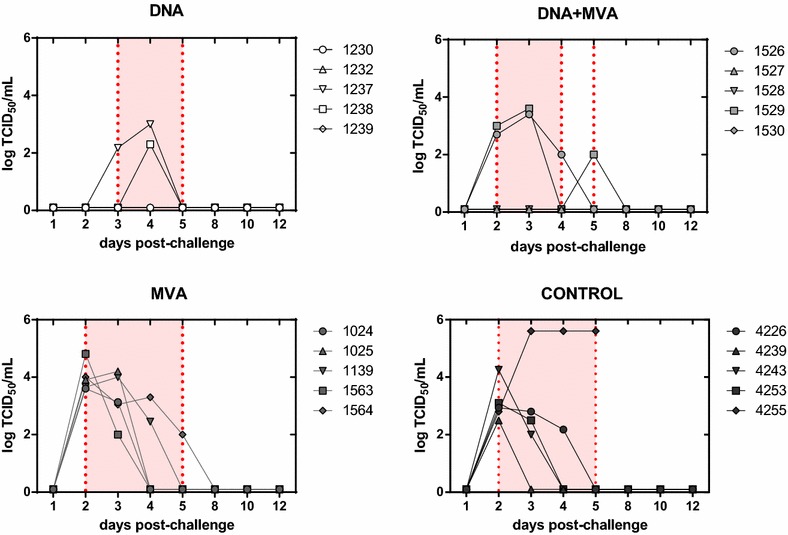
**Viremia titers in vaccinated or mock vaccinated sheep upon RVFV challenge.** Viremia was determined by direct virus titration of individual whole blood samples recovered from days 1–12 post-infection. Lysed blood samples (see “Materials and methods” section) were incubated for 6 days in Vero cells seeded in multi-well 96 plates. End point titers were estimated by the Reed and Muench method [25] based on observation of cytopathic effect upon cell staining.

In order to analyze the efficacy of the vaccines in preventing virus replication, the presence of infectious virus in blood samples, collected at different days after challenge, was determined by titration in Vero cell cultures. In all groups, viremia titers were assayed from blood samples taken from day 1 to day 12 post-challenge. Viremia was detected only in two animals from either DNA or DNA + MVA groups whereas all animals from the control and the MVA groups retained infectivity in blood (Figure 2 and Table 1). In all groups viremia was detected as early as day 2 post-challenge with the exception of two animals in the DNA group, where the titers were detected at days 3 and 4 respectively. These data are in good agreement with the observed delay in the onset of fever in this group. The highest viremia titers were reached in the control sheep #4255 that died at day 5 post-challenge. Taken together, the clinical and virological data points out that the efficacy of the DNA and DNA + MVA vaccines, although partial, was superior than the one displayed by the MVA vaccine alone.

### Blood chemistry analysis

**Figure 3:**
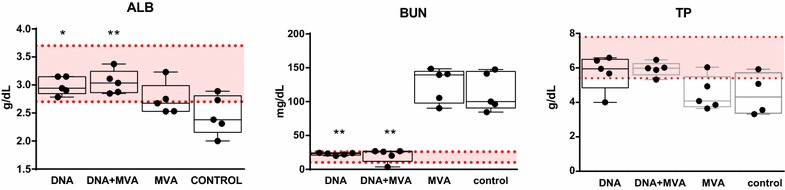
**Biochemical analysis of serum samples obtained from vaccinated or control sheep at day 4 after challenge.** Albumin (ALB), total protein (TP), and blood urea nitrogen (BUN) concentrations measured in serum of individual animals from all groups are depicted. Shadowed area indicates the range for reference (normal) serum values. Asterisks indicate the level of significance with respect to control values (*p ≤ 0.05; **p ≤ 0.01).

Blood chemistries were run on all sera collected at different time points post-infection. Of nine biochemical parameters assessed, only significant differences between groups were detected in serum albumin (ALB) and blood urea nitrogen (BUN) at day 4 post-infection (Figure 3). Animals from the MVA and control groups showed lower values of ALB and also of total protein (TP), compared with animals from DNA and DNA + MVA groups levels whose values were similar to normal range. BUN, an indicator of renal damage, was found significantly elevated in animals from both MVA and control groups compared with the values obtained in animals from DNA and DNA + MVA groups that were similar to normal range (10.3–26 mg/mL). While some sheep showed some particular hepatic enzyme values out of the normal range these could not be directly attributed to RVF disease. Interestingly, the only animal that showed elevated levels in all hepatic enzymes tested (AST, GGT, ALP and ALT) was #4255 from the control (mock vaccinated) group at day 4 post-challenge. This animal had also high levels of LDH and died by day 5 post-challenge (Additional file 2). These data are in agreement with a lower impact of the RVFV challenge in the sheep vaccinated with either DNA or DNA + MVA strategies.

### Antibody responses upon vaccination and challenge

**Figure 4:**
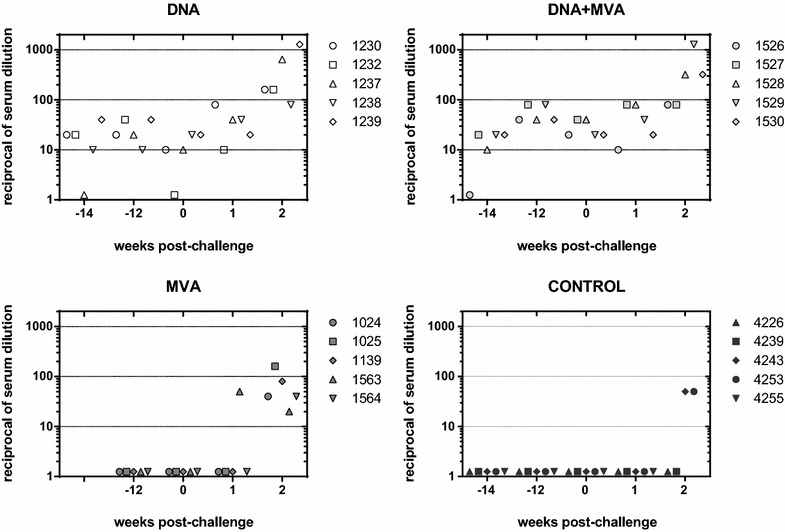
**Neutralizing antibody kinetics.** The neutralizing capability of sera from vaccinated and control (mock vaccinated) sheep sampled at different weeks before or after challenge was tested. The values indicate the reciprocal dilution of serum needed to achieve an 80% reduction of viral plaques (PRNT_80_) in cultured Vero cells.

An indirect ELISA assay was carried out to detect RVFV glycoprotein specific antibodies in sera collected before and after challenge (Additional file 3). While little or no specific antibodies were detected in any of the serum samples before challenge, Gn specific IgGs were detected in all pools from vaccinated groups as early as 15 days post-challenge (week 2 post-challenge), with lower levels in the pool of sera from mock infected sheep. In contrast, Gc specific IgG antibodies were detected during the first week post-infection, with no detectable reactivity in the serum from the control group. A similar response was observed at day 15 post-infection but the highest signal corresponded to the DNA serum pool when compared with the other groups. The presence of RVFV neutralizing antibodies in serum was analyzed by plaque reducing neutralization test (PRNT_80_) (Figure 4). All five sheep vaccinated with the heterologous prime-boost approach showed the highest titers (mean ± SD = 56 ± 19.6) after the last (third) immunization, while the animals that received three doses of DNA showed lower neutralization titers, (mean ± SD = 26 ± 12). In clear contrast, neutralizing antibodies were not detected in sera from the MVA group or mock vaccinated animals. By the time of challenge the titers of neutralizing antibodies in the responsive groups had dropped slightly (mean ± SD of 28 ± 9.8 and 12 ± 7.5 for DNA + MVA and DNA, respectively). During the first week post-challenge (day 5 after challenge) a detectable raise of neutralizing antibodies was found in all sheep from these groups (mean 46 ± 29.3 and 38 ± 24, respectively) while only one sheep from the MVA group was found with elevated neutralizing antibody titers. At day 8 post-challenge (week 2 post-challenge) all animals in the MVA group had developed neutralizing antibodies (mean ± SD = 68 ± 49.9) while only two animals from the control group had neutralizing titers, suggesting that the MVA only vaccinated sheep remained immunologically primed. As expected, higher titers were found in the DNA and DNA + MVA groups (means of 453.7 and 445.1, respectively). These data also confirm that both the DNA and DNA + MVA vaccine approaches are able to prime and induce a faster development of neutralizing antibody responses that can be still detected at least for 12 weeks after immunization.

### Cell mediated immune responses

**Figure 5:**
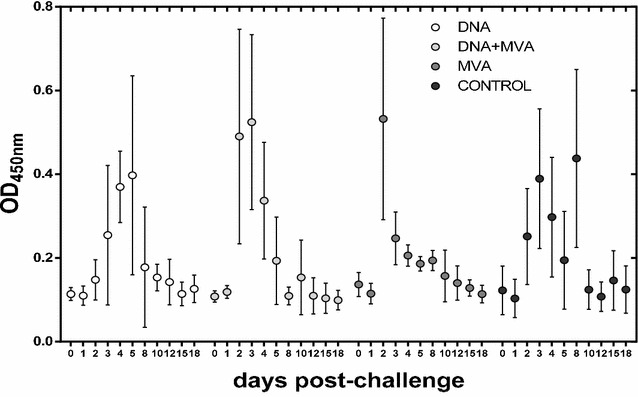
**Detection of plasmatic levels of IFNγ after challenge:** Mean IFNγ capture ELISA values plus standard deviations detected in plasma from vaccinated and control sheep collected at the indicated time points post-infection.

**Figure 6:**
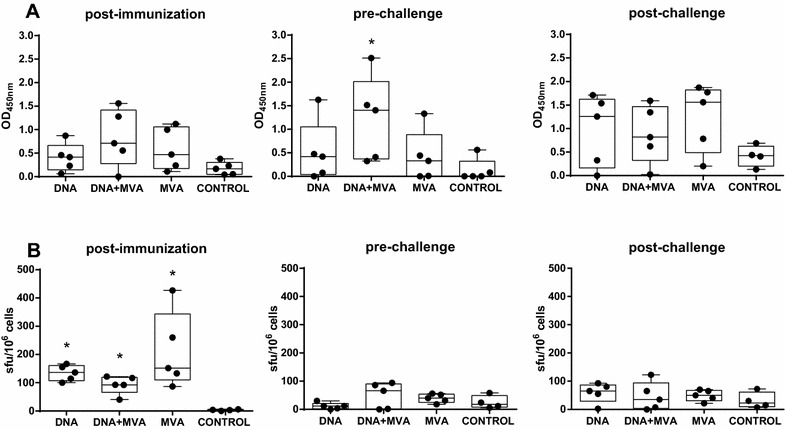
**Detection of secreted IFNγ in plasma after in vitro re-stimulation. A** Whole blood sampled from sheep either after last immunization (post-immunization), just before challenge (prechallenge) or 8 days after challenge (post-challenge) was incubated overnight with RVFV antigen. IFNγ levels in plasma were determined by capture ELISA. **B** Detection of virus specific T-cell responses by IFNγ ELISPOT in vaccinated and control sheep after last immunization, just before challenge or 8 days after challenge. Asterisks indicate the level of significance with respect to control values (*p ≤ 0.05).

The induction of cellular immune responses upon infection among the different vaccine groups was checked at different time points post-challenge using an IFNγ capture-ELISA (Figure 5). Elevated plasmatic levels of IFNγ were detected as early as 2 days post-challenge in samples from sheep vaccinated with the homologous MVA prime/boost (peak at day 2 post-challenge) and the heterologous DNA/MVA approach (peak at days 2–3 post-challenge). In contrast, slower kinetics of IFNγ levels were detected in DNA only vaccinated animals, with a delayed peak between days 4 and 5 post-challenge. Increasing IFNγ levels in the control sheep were also found (starting at day 2 post-challenge) with a maximum at day 3 but with lower magnitude than in vaccinated sheep. Interestingly, two sheep showed a secondary peak at day 8 post-challenge in the control group. This was not detected in any sheep from the vaccine groups. To confirm that the specificity and magnitude of the IFNγ response observed in vivo after challenge was indeed vaccine-induced, the secretion of IFNγ in plasma was measured in blood samples upon in vitro stimulation with RVFV-MP12 antigen. Samples taken either after the last immunization (post-immunization day 38 for DNA and DNA + MVA groups and day 24 for MVA group) or just before challenge (pre-challenge, day 0) were used. A more elevated IFNγ response was observed in vaccinated animals with respect to control sheep. The highest signal was detected in the DNA + MVA group at both times tested, becoming statistically significant at the pre-challenge time when compared to that of the control group (Figure 6A). The production of IFNγ in blood samples at day 8 post-challenge was also tested upon in vitro re-stimulation. Again, only vaccinated animals had elevated plasmatic IFNγ indicative of the priming effect over the control sheep. Furthermore, we detected IFNγ secretion by means of an ELISPOT assay. All vaccinated groups showed a significantly higher number of IFNγ secreting cells when compared to the control group after immunization, although the number of secreting cells had decreased at the time of challenge and was not increased by day 8 post-challenge (Figure 6B). Taken together these results confirm that the different strategies of vaccination used in this study were able to elicit and induce memory cellular responses although with a different magnitude.

## Discussion

Experimental veterinary vaccines tested in laboratory animals with good results in terms of immunity induction and/or efficacy warrant to be further tested in target species. We and others have shown that DNA vaccination and/or recombinant MVA vectors encoding RVFV glycoprotein antigens were able to confer protection against lethal challenge in mouse models by means of distinct immune mechanisms; while DNA vaccination induced more balanced T-helper (Th) responses, the MVA vaccine was more prone to stimulate Th1 responses with little or no detection of neutralizing antibodies [20].

Since the main goal of our investigations was to evaluate RVF vaccine approaches for application in target animal species we then tested whether these vaccines could show efficacy against a RVFV challenge in sheep. Thus we showed that a single administration in sheep of a MVA vaccine encoding RVFV GnGc glycoproteins did not provide complete protection against viremia but it was able to reduce the level of viral excretion. This result warranted further experimentation in ovines in order to improve and extend the magnitude and duration of the prophylactic effect [19]. For this purpose, in this work, we tested the type, quality and duration of the immunity elicited in sheep by DNA and MVA vaccines delivered by both homologous and heterologous (DNA/MVA) prime-boost regimes. Since one of the main goals of vaccination is to provide sustained protective immunity levels in the host, we tested the ability of these vaccination approaches to protect against a delayed virus challenge. This late challenge approach was preferred since it might better assess the effect of field vaccination in an enzootic setting, when circulating virus is present but the moment of infection is unknown, thus helping to make a more accurate interpretation of the real potential of the vaccine. Therefore, to estimate the level of protection provided by these vaccines, sheep were challenged late after vaccination: 12 weeks after the final boost the sheep were inoculated subcutaneously with RVFV and monitored for morbidity for two additional weeks.

Our results show that all three vaccine regimes tested were able to induce an RVFV-specific immune response although with clear differences in terms of efficacy, type and magnitude of the immunity elicited. At the humoral level the immunization with both DNA and DNA + MVA vaccine regimens ensured consistent production of serum with neutralizing activity in vitro, in spite of the apparent lack of reactivity shown in glycoprotein ELISA. In contrast, the MVA-only vaccine approach was not efficient enough in raising neutralizing antibody titers, confirming our previous data in mouse models where neutralizing antibody induction was low or undetectable [20]. Other reports using MVA vaccines encoding viral glycoprotein antigens from Chikungunya virus (CHIKV) have shown divergent outcomes with respect to the induction of virus neutralizing (VN) antibodies, indicating that the expression of each antigen, processing and/or subcellular localization can influence the type of immune response elicited [27, 28]. Apart from the intrinsic immunogenicity of each particular vaccine antigen, a possible explanation for the poor neutralizing antibody response may be related with the properties of the MVA vaccine preparation used in these experiments. Since we used sucrose purified MVA virus particles the immunity elicited can be attributed mostly to the expression and intracellular processing of the recombinant antigen generated upon a non-productive infection of the host cells. Other authors have reported recently that the amounts of pre-formed recombinant antigens in the inoculum can greatly influence the level of humoral response [29]. Thus, for African Horse Sickness Virus (AHSV), when an MVA-AHSV-VP2 infected cell lysate was used for vaccination of mice, high VN titers where elicited, while after vaccination with a sucrose purified preparation from the same cell lysates the induction of neutralizing antibodies was totally abrogated [30]. Therefore, important differences in the humoral immunogenicity could be also attributed to the degree of purification of the MVA vaccine preparations.

It is generally assumed that the presence of neutralizing antibodies is the main correlate of protection against RVFV. In spite of the humoral immunogenicity provided by two of the vaccine strategies using DNA for priming, a decrease in the level of neutralization titers was evident by the day of challenge with respect to the level achieved after the last vaccination dose. Nonetheless, in both groups detectable levels of neutralizing antibodies were present, perhaps explaining the reduced number of viremic animals (2 out of 5) as well as the delayed onset and reduced viremia titers observed with respect to the control or MVA only groups. It appears therefore that the induction of a specific and durable neutralizing antibody response guarantees efficacy. In this sense, the neutralizing antibody titers elicited by the heterologous prime-boost approach were superior than those obtained by homologous DNA prime-boost although these titers remain below those obtained by attenuated or other vector based RVFV vaccine platforms [31–34]. Therefore an obvious improvement of our vaccine approaches would be to increase the magnitude of the neutralizing antibody response.

With respect to the induction of cellular immunity vaccines including MVA in the formulation were superior IFNγ inducers. Particularly, the combined vaccine approach (DNA/MVA) induced both higher and sustained cellular responses, probably due to the booster effect of the MVA vector, known to greatly stimulate T cell responses. There are few reports on the relevance of memory cell responses against RVFV in sheep since most of these studies have focused on the humoral neutralizing antibody responses [35, 36]. Our data show that the induction of cellular responses in the absence of neutralizing antibodies (provided by the homologous MVA prime-boost) may not suffice to ensure a prolonged protective effect long after vaccination since even though the levels of neutralizing antibodies and IFNγ appeared to raise early after challenge, the clinical and virological data were far less favorable in terms of efficacy. Similar to what was observed previously with a single MVA vaccination in sheep, our data reveals a failure of the homologous MVA approach in mounting a robust and durable immune response able to reduce the clinical outcome and blocking virus replication in the host.

An important aspect that may influence the induction of immune responses by MVA vaccines in ruminants is the route for vaccine delivery. We used the subcutaneous route for MVA delivery into sheep after needle injection. Parenteral administration (either subcutaneous, intramuscular or intradermal delivery) results in the interaction of vaccine with dendritic cells (DC) draining the skin through lymphatic vessels and promoting antigen presentation to naïve T-lymphocytes in the lymph nodes. In previous immunizations made with MVA vaccine vectors in ruminants, it was reported the induction of DC apoptosis upon MVA infection, reducing the subsequent induction of T-cell responses [37]. Interestingly, this phenomenon was not as apparent in mice or human DCs. Therefore, it may be interesting to investigate whether the MVA vector, which has proven success in humans and mouse models, might not be suitable as it stands for ruminants. Although the MVA vector has lost an important proportion of host-range genes it still retains a formidable coding capacity that may be more detrimental for species other than mouse and humans in terms of eliciting immune responses. In this sense, ways for the improvement of recombinant MVA vaccines in ruminants have been proposed, for example by reducing their capacity to induce apoptosis of dendritic cells [37].

The adult sheep RVFV infection model may result problematic to ensure reproducibility of disease outcomes among individuals [38]. In our hands, animals that were challenged without previous vaccination (control group) showed different disease outcomes after challenge. In this group the mortality was limited to 1 out of 5 sheep and the viremia levels were markedly different in terms of both onset and duration. A potential explanation for these inconsistent outcomes may be related to the amount of virus inoculated per sheep. We used a dose of 10^5^ TCID_50_ per sheep inoculated subcutaneously to facilitate comparison with our previous experimental challenges in sheep. Inoculation of higher doses (up to 10^7^ pfu per animal) can ensure more reproducibility between individuals as shown recently [34, 38]. Secondly, the induction of humoral immune responses upon vaccination clearly differed among sheep, with some individuals showing earlier and more durable induction of neutralizing antibodies and others with little response within the same vaccination groups. These differences could perhaps be due to the actual dose of vaccine administered. Since doses proved to protect mice were also high (10^7^ pfu/mouse for MVA and 100 µg/mouse for DNA) it could be possible that the vaccine doses used in our sheep trial (10^8^ pfu of MVA/sheep and 400 µg of DNA/sheep) were still below an optimal protective threshold. Both considerations together with the outbred status of the sheep breed used can explain the outcome of heterogeneous data. Therefore, further studies should perhaps rely in stronger indicators for vaccine efficacy such as the ability to avoid abortion in ewes as has been reported for different vaccine approaches [12, 14, 31, 39–43]. In summary, our data suggest that while the use of a MVA-only based vaccine may not be a suitable approach to provide efficacy against RVF in sheep, DNA priming contributes to control the acute phase of the infection by inducing a neutralizing antibody response. This promising result opens the way to further research in order to improve the magnitude of the protective response elicited.

## Additional files

**Additional file 1.** **Clinical findings in lambs.** Clinical evaluation was performed daily for 2 weeks after challenge. The extent of morbidity was quantified according to the presence of different signs of morbidity: nasal and/or ocular discharge, anorexia, diarrhea, prostration and weakness. For each sheep the graphs display the sum of different clinical signs observed at different days post-challenge.

**Additional file 2.** **Blood chemistry values of liver transaminases, total bilirrubin and lactate dehydrogenase in individual sheep at 4** **days post-challenge.**

**Additional file 3.** **Detection of specific RVFV glycoprotein antibodies.** An indirect ELISA assay was carried out to detect Gn and/or Gc specific antibodies in sera from vaccinated and mock-vaccinated animals. Plates were coated with purified recombinant Gn and Gc ectodomains expressed in Schneider’s insect cells [44].
