# Supplementary figures and images for: Efficacy of different DNA and MVA prime-boost vaccination regimens against a Rift Valley fever virus (RVFV) challenge in sheep 12 weeks following vaccination
Source: Vet Res. 2018 Feb 21;49:21. doi: 10.1186/s13567-018-0516-z (PMC5822472; doi:10.1186/s13567-018-0516-z)

## Slide 1
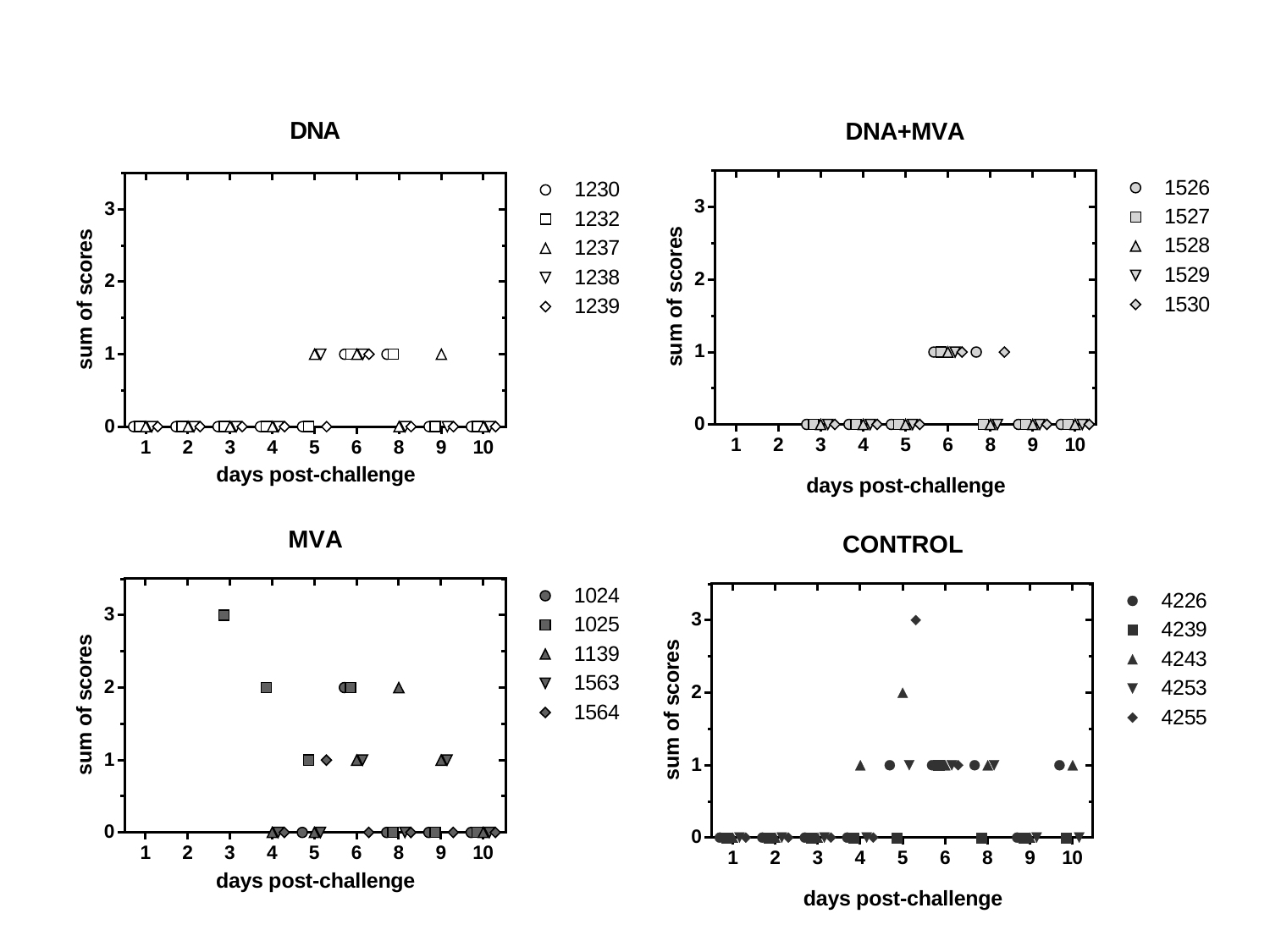

Supplement: Supplementary file 1 — Additional file 1. Clinical findings in lambs. Clinical evaluation was performed daily for 2 weeks after challenge. The extent of morbidity was quantified according to the presence of different signs of morbidity: nasal and/or ocular discharge, anorexia, diarrhea, prostration and weakness. For each sheep the graphs display the sum of different clinical signs observed at different days post-challenge. [file 13567_2018_516_MOESM1_ESM.pptx]

## Slide 1
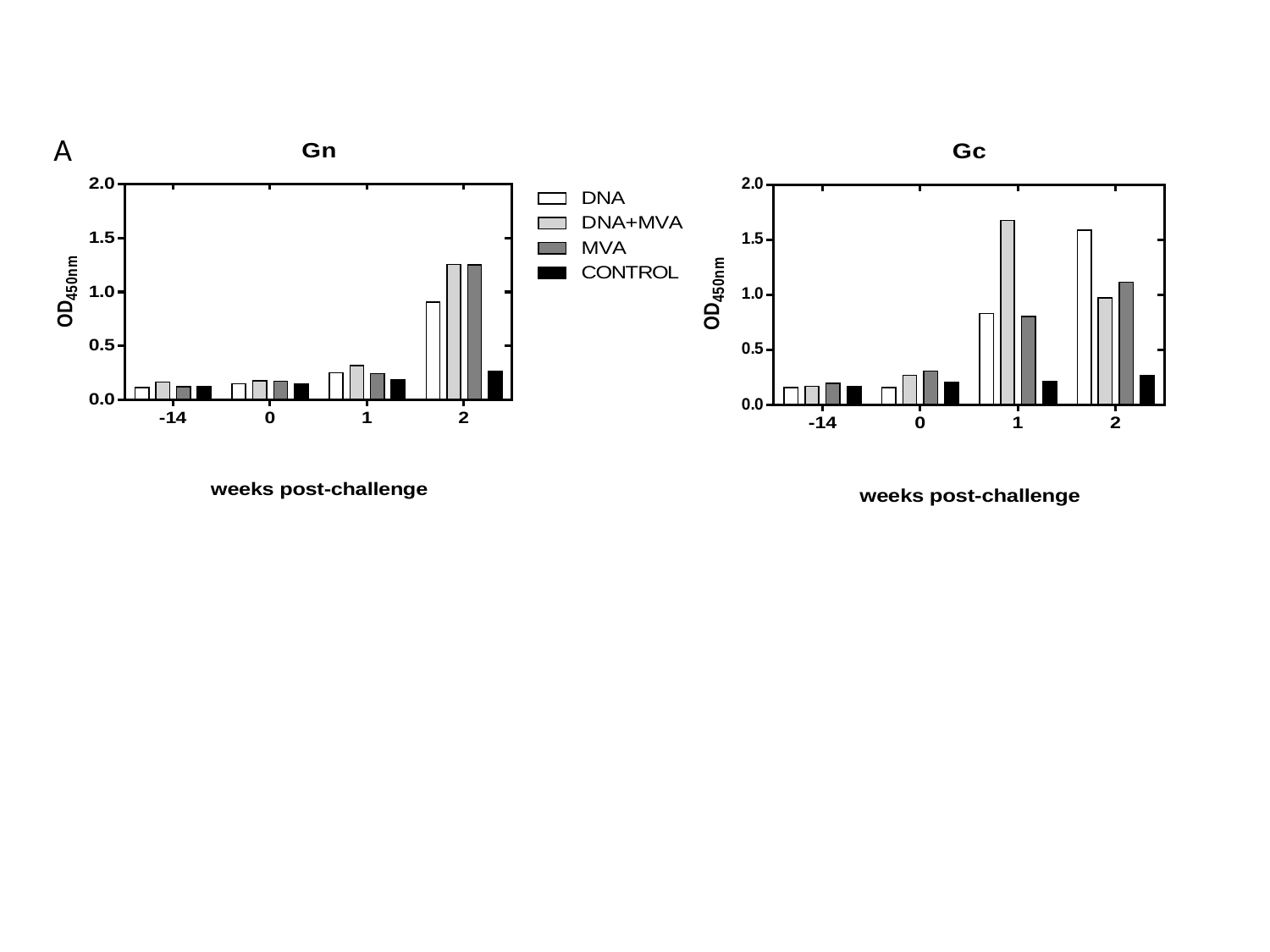

A

Supplement: Supplementary file 3 — Additional file 3. Detection of specific RVFV glycoprotein antibodies. An indirect ELISA assay was carried out to detect Gn and/or Gc specific antibodies in sera from vaccinated and mock-vaccinated animals. Plates were coated with purified recombinant Gn and Gc ectodomains expressed in Schneider’s insect cells [44]. [file 13567_2018_516_MOESM3_ESM.pptx]
