# Supplementary material for: Efficacy of different DNA and MVA prime-boost vaccination regimens against a Rift Valley fever virus (RVFV) challenge in sheep 12 weeks following vaccination
Source: Vet Res. 2018 Feb 21;49:21. doi: 10.1186/s13567-018-0516-z (PMC5822472; doi:10.1186/s13567-018-0516-z)
Supplement: Supplementary file 2 — Additional file 2. Blood chemistry values of liver transaminases, total bilirrubin and lactate dehydrogenase in individual sheep at 4 days post-challenge. [file 13567_2018_516_MOESM2_ESM.docx]

| Sheep number | Vaccine group | **ALP**  [70-390 U/L]^a^ | **AST**  [60-280 U/L]^a^ | **GGT**  [20-52 U/L]^a^ | **ALT**  [22-38 U/L]^a^ | **BIL**  [0-0.27mg/dL]^a^ | **LDH**  [676-1341 U/L]^a^ |
| --- | --- | --- | --- | --- | --- | --- | --- |
| 1230 | DNA | 152.8 | 56,8* | 58* | 6* | 0,22 | 620* |
| 1232 | DNA | 229.5 | 64,8 | 90* | 8* | 0,16 | 830 |
| 1237 | DNA | 66.4* | 123 | 50 | 16 | 0,22 | 882 |
| 1238 | DNA | 181.1 | 170,2 | 99* | 22 | 0,19 | 1208 |
| 1239 | DNA | 385.8 | 117,8 | 100* | 13* | 0,19 | 1143 |
| 1526 | DNA+MVA | 174.2 | 354,7* | 85* | 32 | 0,16 | 2074* |
| 1527 | DNA+MVA | 243.9 | 115,7 | 78* | 18* | 0,06 | 1070 |
| 1528 | DNA+MVA | 185 | 134,6 | 81* | 11* | 0,16 | 1328 |
| 1529 | DNA+MVA | 153.6 | 183,8 | 56* | 12* | 0,16 | 918 |
| 1530 | DNA+MVA | 147.2 | 139,6 | 84* | 20* | 0,1 | 933 |
| 1024 | MVA | 185.4 | 160,7 | 83* | 24 | 0,11 | 1290 |
| 1025 | MVA | 168.4 | 189,1 | 169* | 25 | 0,01 | 1495* |
| 1139 | MVA | 137.7 | 247,9 | 1* | 24 | 0 | 2176* |
| 1563 | MVA | 191 | 143,2 | 93* | 26 | 0,27 | 1090 |
| 1564 | MVA | 237.5 | 170,4 | 2* | 21* | 0,11 | 964 |
| 4226 | control | 293 | 223,9 | 82* | 26 | 0,14 | 1012 |
| 4239 | control | 141.1 | 131,3 | 101* | 17* | 0,08 | 1024 |
| 4243 | control | 141.1 | 64,6 | 84* | 11* | 0,14 | 746 |
| 4253 | control | 142.6 | 109,1 | 0* | 13* | 0,29* | 1039 |
| 4255 | control | 394.6* | 622,8* | 304* | 114* | 0,08 | 5522* |

^a^reference values given in brackets as in [[26](#_ENREF_26)], *outliers
